# Neighborhood Cognability and Cognitive Performance Approaching Midlife in the CATSLife Study

**DOI:** 10.1093/geroni/igaf122.1674

**Published:** 2025-12-31

**Authors:** Chandra Reynolds, Jessica Finlay, Elizabeth Muñoz, Robin Corley, Soo Rhee, Sally Wadsworth

**Affiliations:** University of Colorado Boulder, Boulder, Colorado, United States; University of Colorado Boulder, Boulder, Colorado, United States; University of Texas at Austin, Austin, Texas, United States; University of Colorado Boulder, Boulder, Colorado, United States; University of Colorado Boulder, Boulder, Colorado, United States; University of Colorado Boulder, Boulder, Colorado, United States

## Abstract

Neighborhood features may support cognitive health in later life through social and behavioral pathways such as physical activity, social connection, and cognitive engagement, as well as reduced exposure to pollution and fast-food. Whether such neighborhood features—conceptualized as Cognability—are associated with cognitive functioning before midlife is unknown. In the present study, we evaluated Cognability and cognitive performance associations at midlife and tested for possible selection effects by controlling for adolescent cognitive performance. We analyzed data from 1061 participants from the Colorado Adoption/Twin Study of Lifespan behavioral development and cognitive aging (CATSLIfe; Mage= 33 years, range 28-49; 53% female, 10% non-white/Hispanic; tested between 2015-2021) with available year 16 IQ assessments. Cognability scores (2017) and Social Vulnerability Index SES scores (SVI-SES, 2018) were aligned. WAIS-III IQ scores indexed cognitive performance. In generalized estimating equations, we regressed current IQ on Cognability, adjusting for SVI-SES, age, sex, race/ethnicity, adoption status, and sibling similarity, then tested whether entering adolescent IQ changed the observed associations. Results revealed that higher neighborhood Cognability was associated with higher full-scale IQ at midlife (B = 0.9894, p = 0.0100), even when adjusting for neighborhood SES and individual sociodemographics. However, the Cognability–IQ association was attenuated by 74% when adolescent IQ was entered (B = 0.2537, p = 0.2249). Similar patterns were observed for verbal and performance IQ. We observed positive associations between neighborhood Cognability and IQ at midlife even when adjusting for current neighborhood SES and individual sociodemographics. However, neighborhood selection may partly underlie these associations, as accounting for adolescent IQ diminished the associations.

